# Mitochondrial Dysfunction in Aging and Age-related Disorders

**DOI:** 10.14336/AD.2025.10731

**Published:** 2025-07-31

**Authors:** Aida Adlimoghaddam

**Affiliations:** ^1^Department of Neurology, Dale and Deborah Smith Center for Alzheimer's Research and Treatment, Neuroscience Institute, Southern Illinois University School of Medicine, Springfield, Illinois, USA.; ^2^Department of Pharmacology, Southern Illinois University School of Medicine, Springfield, Illinois, USA.; ^3^Department of Medical Microbiology, Immunology and Cell Biology, Southern Illinois University School of Medicine, Springfield, Illinois, USA.

**Keywords:** Mitochondria, Energy metabolism, Aging, Age-related Disorders, Neurodegenerative Disorders

## Abstract

Mitochondrial dysfunction is increasingly recognized as a unifying mechanism underlying aging and a wide range of age-related diseases. This special issue brings together recent advances that elucidate how impaired mitochondrial function contributes to neurodegenerative, cardiovascular, and metabolic disorders. The featured articles highlight molecular pathways of mitochondrial decline, its systemic consequences, and potential interventions aimed at restoring mitochondrial health. Collectively, these studies reinforce the concept that targeting mitochondrial integrity holds significant promise for promoting healthy aging and preventing chronic disease.

## Mitochondrial Dysfunction and Aging: A Multisystem Challenge

As cellular powerhouses, mitochondria maintain energetic homeostasis, regulate oxidative stress, and modulate apoptosis. Age-related mitochondrial decline manifests across multiple biological processes, including impaired oxidative phosphorylation (OXPHOS), elevated reactive oxygen species (ROS), altered mitochondrial dynamics, and compromised mitophagy [1-3]. Several studies in this issue illuminate how these defects contribute to disease pathogenesis, particularly in the brain and cardiovascular system.

For example, Zhang et al. present a comprehensive roadmap linking mitochondrial dysfunction to cardiovascular aging, revealing that oxidative stress, disrupted dynamics, and mitochondrial DNA (mtDNA) damage underlie vascular and cardiac decline in aging individuals [4]. Similarly, Yuan et al. discuss how early-life mitochondrial interventions may enhance longevity and resilience against age-related degeneration [5].

Beyond energy production, mitochondria regulate apoptosis, amino acid and lipid metabolism, and calcium homeostasis, all of which become dysregulated with age. In neurodegenerative disorders, mitochondrial dysfunction is associated with iron and calcium imbalance, decreased mitochondrial mass and membrane potential, defective mtDNA, increased ROS, and abnormalities in mitophagy, bioenergetics, and trafficking.

## Neurodegeneration and the Mitochondrial Axis

Mitochondrial dysfunction is a hallmark of Alzheimer’s disease (AD), Parkinson’s disease (PD), and Huntington’s disease (HD). Several articles in this issue underscore the intersection between mitochondrial biology and neurodegeneration. For instance, Mohan and Kumar explore TCA cycle impairment in AD and its cascading effects on energy metabolism and cognitive decline [6].

Pokotylo et al. analyze non-OXPHOS energy pathways in PD, proposing novel metabolic targets for therapeutic modulation [7]. Dagda and colleagues further highlight mitochondrial dysfunction in PD as both a driver and target of disease pathology [8].

Oxidative damage and iron dyshomeostasis are also implicated in ferroptosis, a regulated cell death process recently linked to AD. Bai et al. examine how iron metabolism and ferroptosis contribute to neurodegeneration through a dual-pathway mechanism [9]. Tang et al. expand this theme by detailing the role of protein S-nitrosylation in regulating mitochondrial quality control mechanisms in CNS diseases, emphasizing its therapeutic potential [10].

Interestingly, mitochondrial dysfunction may occur in a sex-dependent manner. Females often exhibit earlier signs of mitochondrial impairment than males. Estrogen, which modulates mitochondrial gene expression and function, may play a protective role. Its decline with aging could increase women’s vulnerability to neurodegeneration, suggesting that hormone-based mitochondrial therapies may require sex-specific tailoring.

## Quality Control and Inter-Organelle Communication

Loss of mitochondrial proteostasis and disrupted inter-organelle communication accelerates aging and disease progression. Hu et al. demonstrate that presenilin regulates contacts between mitochondria and lysosomes in *C. elegans*, a process essential for autophagic clearance and cellular longevity [11].

These quality control mechanisms are further impaired in various models of mitochondrial damage, revealing a central vulnerability in neurodegenerative pathology. Excitotoxicity and ferroptosis-related pathways also converge on mitochondrial targets, opening novel therapeutic windows [12].

## Therapeutic Strategies: From Antioxidants to Transplantation

A range of mitochondrial-targeted therapies are under investigation, including gene therapies, redox-based interventions, and cationic compounds such as thiobutyl-triphenylphosphonium (Thiobutyl-TPP). Dimebon has shown cognitive benefits in AD and HD models but awaits large-scale clinical validation [13].

Adlimoghaddam et al. describe mitochondrial transfusion (MT) as a promising therapeutic strategy. This approach involves introducing healthy mitochondria into damaged cells and has been shown to enhance respiratory capacity, reverse immune senescence, and improve survival in infection models using platelet-derived mitochondria or “mitlets” [14,15]. These innovations mark a shift toward bio-restorative approaches in mitochondrial medicine.

Exercise is also a potent modulator of mitochondrial biogenesis and function. Chen et al. report that various physical activity modalities activate the PKG-STAT3-Opa1 axis, thereby enhancing cardiac mitochondrial performance [16].

## Mitochondrial Dysfunction Beyond the Brain

Mitochondrial dysfunction extends beyond the nervous system to impact multiple organ systems. In Down syndrome, Xu et al. report that mitochondrial imbalance may accelerate brain aging [17]. In ophthalmology, Wang et al. explore mitochondrial energy failure in optic disc drusen [18]. Other studies in this issue investigate mitochondrial roles in sarcopenia, immune aging, blood-brain barrier dysfunction, and cognitive resilience—broadening the landscape of mitochondrial medicine.

## Conclusion

This expanded editorial integrates key findings across the special issue, offering a panoramic view of how mitochondrial dysfunction drives aging and disease. The evidence affirms mitochondria as both sentinels and mediators of systemic health. With over 300 mitochondrial disorders now recognized, investment in mitochondrial biology is no longer optional—it is essential. As mitochondrial medicine evolves, it holds the potential not only to extend lifespan but to enhance healthspan for aging populations worldwide.
